# Misorientation-angle-dependent electrical transport across molybdenum disulfide grain boundaries

**DOI:** 10.1038/ncomms10426

**Published:** 2016-01-27

**Authors:** Thuc Hue Ly, David J. Perello, Jiong Zhao, Qingming Deng, Hyun Kim, Gang Hee Han, Sang Hoon Chae, Hye Yun Jeong, Young Hee Lee

**Affiliations:** 1 IBS Center for Integrated Nanostructure Physics (CINAP), Institute for Basic Science, Sungkyunkwan University, Suwon 440-746, Korea; 2 IFW Dresden, Institute of Solid State Research, P.O. Box 270116, D-01171 Dresden, Germany; 3 Department of Energy Science, Sungkyunkwan University, Suwon 440-746, Korea

## Abstract

Grain boundaries in monolayer transition metal dichalcogenides have unique atomic defect structures and band dispersion relations that depend on the inter-domain misorientation angle. Here, we explore misorientation angle-dependent electrical transport at grain boundaries in monolayer MoS_2_ by correlating the atomic defect structures of measured devices analysed with transmission electron microscopy and first-principles calculations. Transmission electron microscopy indicates that grain boundaries are primarily composed of 5–7 dislocation cores with periodicity and additional complex defects formed at high angles, obeying the classical low-angle theory for angles <22°. The inter-domain mobility is minimized for angles <9° and increases nonlinearly by two orders of magnitude before saturating at ∼16 cm^2^ V^−1^ s^−1^ around misorientation angle≈20°. This trend is explained via grain-boundary electrostatic barriers estimated from density functional calculations and experimental tunnelling barrier heights, which are ≈0.5 eV at low angles and ≈0.15 eV at high angles (≥20°).

## Abstract (web summary)

Grain boundaries can degrade the performance of electronic devices made from single atomic layers of transition metal dichalcogenides. Here, the authors combine transport measurements and transmission electron microscopy to find a correlation between field-effect mobility and grain misorientation angle.

## Graphical abstract

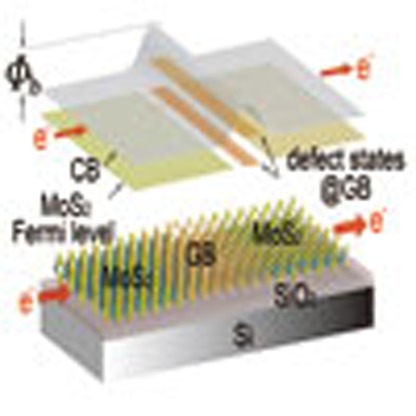

Understanding the atomic structures of grain boundaries (GBs) is essential to controlling and improving electrical transport properties in both bulk and low-dimensional materials. In traditional mesoscopic semiconductors, GBs are scattering sites for majority carriers and degrade transport via the formation of electrostatic barriers^1^,^2^. Such degradation is amplified in low-dimensional materials due to the limited phase space and reduction in Coulomb screening^3^. Two-dimensional (2D) transition metal dichalcogenides are one class of low-dimensional materials with unique electrical and electro-optical properties showing promise for emerging valleytronics^4^ and soft electronics applications^5^. However, large-area monolayer transition metal dichalcogenides synthesized via chemical vapour deposition (CVD) inevitably possess GBs between randomly oriented crystal domains. Early theoretical research involving density functional theory (DFT) calculations for MoS_2_ predicted the possibility of both metal and semiconductor GB characteristics, depending on the local atomic structures^6^,^7^,^8^. However, experimental assessment of MoS_2_ GB transport is still under debate in the literature^6^,^7^,^8^,^9^,^10^,^11^ due to a large device-to-device performance variation, poor single-domain carrier mobility, and, most importantly, lack of correlation between transport properties and GB atomic structures.

Here, we overcome these difficulties by directly correlating four-probe transport measurements across single GBs with both high-resolution transmission electron microscopy (TEM) imaging of the measured devices and first-principles calculations. Using an inert-environment fabrication procedure, we observe a positive and nonlinear correlation between field effect mobility (*μ*_FE_) and misorientation angle (*α*). The precise boundary location, orientation and atomic structure of transistor devices were also examined by TEM, results of which were cross-referenced with further characterization and imaging of pristine MoS_2_ GBs. On using DFT to calculate the *α*-dependent local GB band structures, equilibrium electrostatic GB barriers were found to be correlated with experimentally determined barriers, which gives a clear understanding of the underlying mobility dependences.

## Results

### Electrical characterization

Monolayer MoS_2_ flakes (typical sizes of 20–40 μm) were grown by CVD and subsequently transferred onto SiO_2_ (100 nm)/Si substrates (see Methods). GB positions were then initially determined by dark-field optical microscopy images while considering the growth kinetics (Supplementary Fig. 1). Regions with no sign of wrinkling or multilayers were then selected to prevent misinterpretation of the transport mechanism. To suppress interaction with oxygen-related functional groups, further device fabrication was performed in a glove box (H_2_O, O_2_<1 p.p.m.) and only acetone solvent was used during the electron-beam lithography procedures to minimize additional GB functionalization.

**Figure 1:**
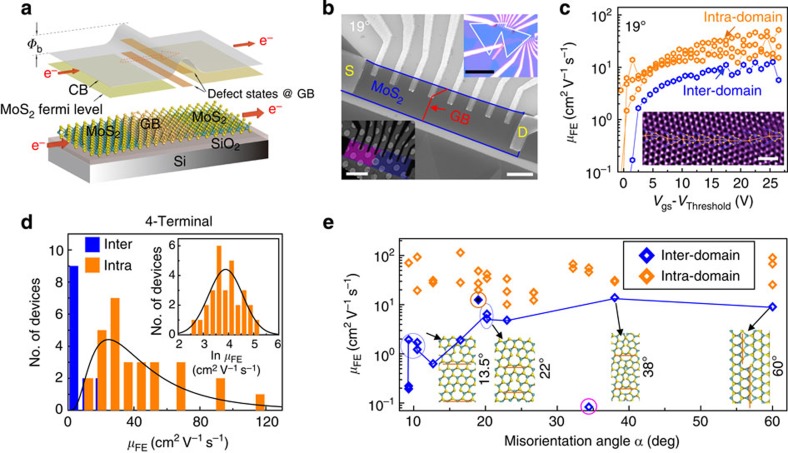
Measurements and statistics on Intra-domain vs inter-domain transport. (**a**) Experimental schematic of carrier transport across a grain boundary. (**b**) SEM image of a device sample on SiO_2_/Si wafer; scale bar, 3 μm. After device characterization, the device was transferred to a TEM grid; scale bar, 6 μm (left inset). Right inset: Optical micrograph of the device with pre-etch flake shape outlined; scale bar, 30 μm. (**c**) Electrical performance of the device in panel (**b**) with *α*=19°. Intra-domain regions always have a higher *μ*_FE_ and reduced *R*_S_ when compared to inter-domain. An STEM image of the boundary is shown in the inset. Inset scale bar, 1 nm. (**d**) Statistical distribution of the intra- and inter-domain mobilities with *μ*_FE(inter)_<16 cm^2^ V^−1^ s^−1^<*μ*_FE(intra)_. The intra-domain *μ*_FE_ displays a log-normal distribution (black line) with expectation value *μ*_FE_=44 cm^2^ V^−1^ s^−1^, as exemplified by ln(*μ*_FE_) in the inset. (**e**) The *α*-dependent *μ*_FE(inter)_ (blue) for 10 different angles with *μ*_FE(intra)_ (total of 43 devices). Here, *μ*_FE(inter)_ increases with a rise in *α* from 9° to 20°, but saturates for *α*>20° at *μ*_FE(inter)_<16 cm^2^ V^−1^ s^−1^. The 19° dislocation is of the ‘Down'-type, while the others are ‘Up'-type.

*V*_gs_-dependent transport measurements were performed on an elongated Hall-bar geometry to allow single-crystal and GB four-probe sheet resistance (*R*_s_) and *μ*_FE_ comparison. Figure 1a shows the schematic of a typical carrier transport across a GB, while a representative multi-terminal device tested in this work is displayed in Fig. 1b. A GB with an optical microscopy-determined misorientation angle of 19° is located near the centre of the monolayer MoS_2_ film (red-dashed line), where the white dashed lines (inset) denote the pre-etch shape of MoS_2_ flakes (Supplementary Fig. 2). The *R*_s_ and *μ*_FE_ associated with intra-domain and inter-domain transport were measured as a function of *V*_gs_ by choosing appropriate electrodes either within the domain or across the GB line, respectively (Fig. 1c and Supplementary Fig. 7a). The selected-area electron-diffraction (SAED) pattern (right inset of Supplementary Fig. 3b) and scanning (STEM) findings of the exact device in Fig. 1b both revealed that *α*=19°, which further agrees with the optical estimates within a statistical error of ±2° (Supplementary Fig. 3). In this 19° sample, the intra-domain (inter-domain) *μ*_FE_=24–60 (16) cm^2^ V^−1^ s^−1^ and *R*_s_=30–85 (95) kΩ sq^−1^. After completing the electrical measurements, the devices were expeditiously transferred to an Au TEM grid (an Au grid was used to suppress additional oxidation of the sample as the moisture level should be minimized to prevent sample damage). STEM analysis of the boundary revealed that the GB line consists of a series of 5-7 membered ring cores, as indicated by the orange-dashed line in the inset of Fig. 1c. (Mo atoms are denoted in blue, while S atoms are displayed in yellow.)

For further angle-dependent transport analysis, a statistical distribution of the mobility was obtained from 43 devices (29 intra-domain and 14 inter-domain, refer to Supplementary Fig. 3 for raw mobility data), as shown in Fig. 1d. A decisive cutoff separates intra-domain and inter-domain data at *μ*_FE_=16 cm^2^ V^−1^ s^−1^. The intra-domain *μ*_FE_ dataset forms an ordered but highly dispersive statistical distribution with a minimum (maximum) of *μ*_FE_=17 (115) cm^2^ V^−1^ s^−1^, which results from conduction band-tail states localized near the conduction band edge^12^ (Supplementary Fig. 5). Figure 1d shows the raw data and natural-log of *μ*_FE_ histograms (inset); excellent fitting to a log-normal distribution was observed, with an expected *μ*_FE_=44 cm^2^ V^−1^ s^−1^. Notably, seven devices had a *μ*_FE_>75 cm^2^ V^−1^ s^−1^ and on-state *R*_s_ was consistent with the best-reported exfoliated results^13^ (also see Supplementary Fig. 6).

With statistically reliable intra-domain mobility data to contrast, the inter-domain misorientation angle-dependent mobility was measured. Results are shown in Fig. 1e (*R*_s_ data are presented in Supplementary Fig. 7). Interestingly, the inter-domain mobility increases for small angles (8–20°), although some variability exists in the low-angle data. Above 20°, *μ*_FE_ saturates below the 16 cm^2^ V^−1^ s^−1^ intra-domain cutoff. Overall, the inter-domain mobilities vary by 2 orders of magnitude across the tested range of *α*. It was further observed that although devices with 8–20° inter-domain misorientation display poor transport, these samples consistently conduct, as demonstrated by the large number of inter-domain data points within this range. Conversely, samples within the 20–60° range show better transport properties; many GBs were insulating (broken) due to a torn/poor interface. One example is the 34° sample (Fig. 1e), which was measurable only after annealing at 420 K, and then quickly became insulating and unmeasurable at 300 K. Other missing inter-domain data of Fig. 1e resulted from similar insulating behavior, likely due to tearing during substrate transfer (Supplementary Fig. 8). This observation agrees with previous experiments on 3D crystals, where high-angle samples frequently shattered after growth^14^. The peculiarly high mobility of the inter-domain 19° device results from the boundary crossing the corner of the contact (Fig. 1b,c), which was discovered during post-transfer scanning electron microscopy or STEM analysis.

### Structural characterization

**Figure 2:**
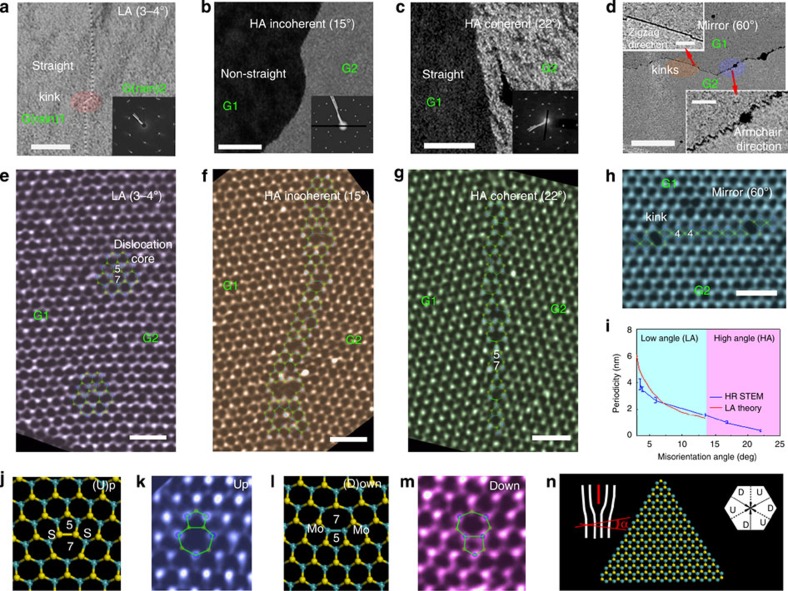
TEM characterization of the MoS_2_ GBs showing misorientation angle dependence. (**a**–**d**) DF-TEM images of low-angle (3-4°), high-angle incoherent (15°)/coherent (22°) and mirror GBs (60°); scale bar, 50 nm. The insets in (**a**-**c**) show SAED patterns of the GBs, while the two inset images in (**d**) display zigzag (upper part) and armchair direction (lower part) mirror GBs. The scale bars in the latter two insets are 20 nm. (**e**–**h**) High-resolution ADF-STEM images correlated to the four cases presented in (**a**–**d**); false colour was applied. Scale bar, 1 nm. The dislocation cores and GBs were superimposed with atomistic models; blue and yellow circles denote Mo and S atoms, respectively, while the two domains are marked as G1 and G2. (**i**) Experimentally measured periodicity of the dislocation cores vs. misorientation angle with LA theory fitting. (**j**–**m**) Two types of LA-GBs: ‘Up' type (S-S bond) and ‘Down' type (Mo-Mo bond) (5-7) dislocation cores. The DFT-optimized models for each dislocation type are shown in (**j**,**l**), while ADF images superimposed with atomistic models are displayed in (**k**,**m**). (**n**) Atomistic model for a triangular flake with Mo-terminated zigzag edges, the upper left inset shows the ‘Up' type dislocation scheme, while the upper right inset shows the six armchair directions in MoS_2_ with either ‘Up' or ‘Down' type dislocations. The directions of all schemes are correlated and aligned.

To directly correlate the transport results to the atomic defect structures, we performed extensive TEM/STEM analysis of the aforementioned devices and many other representative samples; the results are summarized in Fig. 2. The diffraction contrast in the dark field (DF)-TEM images (Figs 2a–d) clearly reveals the morphology and location of the GBs. Low angle (LA)-GBs (0–13°) are rather straight, and formed by aligned dislocation cores, visible as dotted lines in the DF images (Fig. 2a). The SAED is presented in the inset of Fig. 2a, while the high-resolution STEM annular dark field (ADF) image is shown in Fig. 2e.

In bulk specimens, LA-GBs are composed of periodically piled edge dislocations. The distance between the edge dislocation cores can be estimated by *d*=**b**/sin(*α*), where **b** is the Burgers vector. The dislocations are composed of ‘Up' or ‘Down' types (Figs 2j–m) that correspond to S–S bonding or Mo–Mo bonding at the core, respectively. Due to the three-fold (not six-fold) symmetry of the MoS_2_ monolayer, a lack of inversion symmetry exists. Such a scenario induces the formation of two types of LA-GBs with the aforementioned ‘Up' or ‘Down' dislocations along the armchair directions, as highlighted in Fig. 2n. All devices investigated for electrical transport were of the ‘Up'-type boundary variety (except for 19°, which is not utilized in the comparison) and thus comparison can be made. Furthermore, the energies associated with ‘Up/Down' dislocations are similar, and both are energetically favourable since they both have the smallest Burgers vector [11-20]. Due to kinetic growth limitations, GBs with overall relative orientation between the armchair directions of MoS_2_ are also composed of [11−20] dislocations (‘Up/Down'). The boundary is decomposed into short armchair-oriented sections and is slightly kinked on the mesoscopic scale (Fig. 2a). For bulk materials, LA-GBs usually imply *α*<6° due to strong interactions between the strain fields generated by adjacent dislocation cores at higher angles^15^. However, basal plane strain relaxation can occur in 2D materials via local rippling or buckling^16^, thereby allowing for an extension of LA-GB theory to larger angles. The periodicity of the dislocation core distance is inversely proportional to *α*, in accordance with the classical LA-GB theory (Fig. 2i and Supplementary Fig. 9), but the range greatly exceeds the classical limit and is extended up to 13°, at which point the GB lines are bent by intense strain fields. Thermodynamically, these LA-GBs should follow a straight line in a direction perpendicular to the Burgers vector (armchair directions in MoS_2_), which is consistent with our observations (Supplementary Fig. 10).

High-angle (HA)-GBs between two domains can be classified as either coherent or incoherent, the former of which refers to GBs that possess a periodic lattice. Using notation from coincident site lattice theory^17^, the most favourable coherent boundaries for monolayer MoS_2_ are Σ7 (22°), Σ13 (28°), Σ19 (13°), and so on (Supplementary Fig. 11). From the results of TEM/STEM characterizations and DFT calculations (Supplementary Fig. 12), we observed that most HA-coherent GBs also follow straight lines composed of continuous 5–7 ring cores, in a manner similar to LA-GBs (Figs 2c,g). Any GB with an *α* that deviates from coherent angles will be an incoherent GB (Fig. 2b). A curved (non-straight) GB can be identified in the atomic scale images (Fig. 2f). When compared to coherent GBs, the incoherent GBs have a larger aperiodic width of defects, whereas the mirror GBs (60° GB) are mostly straight along the zigzag direction and composed of a series of 4–4 cores with few kinks (Figs 2d,h). However, when the mirror GB follows the armchair direction (due to growth kinetics), it is decomposed into a series of 4–8 cores (Supplementary Fig. 13).

In terms of mechanical stability, LA GBs are mostly stable, regardless of the GB type. This explains how we were able to accumulate a large set of data points, as shown in Fig. 1e. However, in the HA region, only coherent GBs with *α*=22°, 38° and 60° were stable. While many samples contained boundaries with intermediate angles, transport data were not collected for these specimens because GBs were easily fragmented during the transfer process. Since incoherent GBs are generally wider with a complex array of defect structures (see Fig. 2f), they are energetically less stable when compared to coherent GBs. This agrees well with the theoretical prediction that the total energy usually increases in response to the misorientation angle^18^.

## Discussion

**Figure 3:**
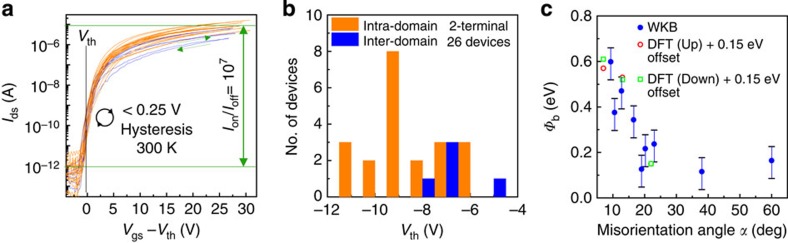
Extraction of Schottky barrier height from the threshold voltage. (**a**) Dual sweep *I*_ds_-*V*_gs_ data for 26 different intra-/inter-domain two-terminal FETs at 300 K. Consistent and trivial 0.25-V clockwise hysteresis was observed for all 26 devices, regardless of the inter-/intra-domain status. Intra-domain devices have an average *I*_on_/*I*_off_=10^7^ at 300 K, while the inter-domain *I*_on_/*I*_off_ was reduced and misorientation angle-dependent. (**b**) Distribution of *V*_th_ from panel (**a**) with a bin size of 0.25 V. The median of the inter-domain *V*_th_ was shifted by +1.6 V from that of the intra-domain. (**c**) Inter-domain tunneling barrier height *Φ*_b_ calculated via JWKB approximation when assuming a parabolic barrier shape and a transmission probability *T*_gb_=*μ*_FE(inter)_/*μ*_FE(intra)_. The error bars are based on one standard deviation of the intra-domain *μ*_FE_.

Dislocations and GBs in III–V systems (e.g., GaN) have previously been modeled by a generalized line charge with density *ρ*_L_ ∝defect density. Increasing *ρ*_L_ induces a larger electrostatic potential and results in a lower effective mobility^19^,^20^. In comparison, here we observe a positive and nonlinear correlation between *μ*_FE_, the dislocation core density and *α*. To explain why greater dislocation core density permits greater inter-domain mobility, and if results are consistent with the aforementioned theoretical models, we first consider whether the boundary is charged. *I*_ds_–*V*_gs_ curves from 26 two-terminal FETs (Fig. 3a) reveal that *I*_off_ is rather invariant and limited by leakage current, while hysteresis is trivial and a uniform 0.25 V (300 K), equal to the *V*_gs_ point spacing. Boundary charge must therefore be fixed, which is further examined by comparing respective *V*_th_ values. As transport is band-tail limited and a well-defined saturation regime is absent in the *I*_sd_–*V*_gs_ curves (Fig. 3a), *V*_th_ is defined by the *V*_gs_ logarithmic turn-on point corresponding to the peak subthreshold swing (SS). This point is denoted at *V*_gs_−*V*_th_=0 by a dotted line in Fig. 3a. We found −11 V<*V*_th_<−5 V for all samples (Fig. 3b), which is comparable to that obtained from STM studies of atomically clean exfoliated samples^21^. A positive 1.6-V shift (in median value) is observed in the inter-domain samples compared with intra-domain, which is clear evidence for the existence of an electrostatic potential barrier. This potential barrier can be estimated by noting that, at *V*_th_, SS∼230 mV dec^−1^. Since 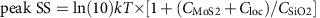 is measured at the Fermi level (*E*_F_) within the bandgap, 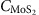
≈0. Substituting 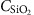 and SS above, we observe that *C*_loc_=76 nF cm^−2^. An order-of-magnitude estimate for the potential barrier height *Φ*_b_ is obtained from an equivalent circuit model with SiO_2_ capacitance in series with the MoS_2_ self-capacitance, 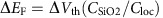
=190 meV (see Methods).

However, in accordance with *μ*_FE_, inter-domain *I*_on_/*I*_off_, and *R*_S_ results (Figs 3a and 1), *Φ*_b_ is *α*-dependent. For 9°*≤α≤*60° the core-density periodicity is less than 2 nm, and thus electrostatic perturbation is essentially a 1D barrier. The *α*-dependence of the effective *Φ*_b_ is calculated via the Jeffreys–Wentzel–Kramers–Brillouin (JWKB) approximation^22^ with an on-state transmission probability (*T*) based on the experimental *μ*_FE(inter)_=*T*_GB_*μ*_FE(intra)_. Assuming a parabolic-shaped potential with a peak magnitude *Φ*_b_ and 8 nm width based on STM observations^23^, the JWKB approximation gives *T*_GB_*=*exp(−8.7*Φ*_b_). Fitting to experimental data, it was found that *Φ*_b_ peaks above 0.5 eV for *α*<10° and then decreases to a saturation point of∼0.15 eV for *α*>20° (Fig. 3c). The error bounds are based on one standard deviation of the intra-domain log-normal distribution (Fig. 1d).

**Figure 4:**
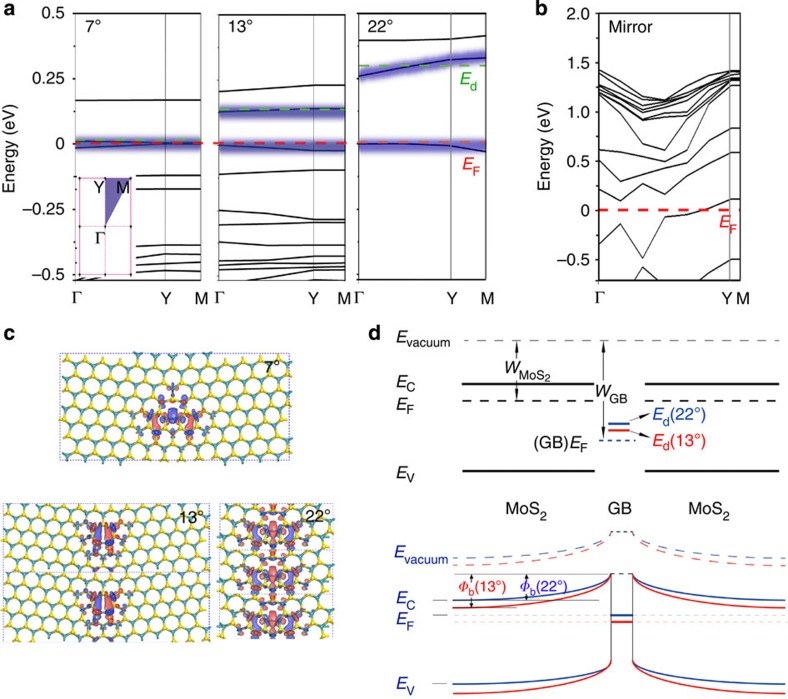
DFT calculations of the band structure for GBs with various misorientation angles. Band structure near the Fermi level (red dash line) of (**a**) tilt and (**b**) mirror GBs. Inset shows the first Brillouin zone. The first band above the Fermi level is highlighted by a green-dashed line. (**c**) The charge densities for the first band above the Fermi level, as indicated by the ‘Down' type of 5–7 dislocation cores for 7°, 13° and 22°. (**d**) The expected flat band alignment near the GB with defect states extracted from the band structures and the corresponding band bending after Fermi level equilibrium. The Schottky barrier height decreases in response to α.

Finally, DFT was used to calculate the band structures of various GBs based on atomic geometries observed in TEM (Fig. 4). Representative coherent GBs of 7°, 13° and 22° were selected (Fig. 4c). The dislocation 5–7 ring core structures for these angles are similar, but a larger bond length variation occurs in the vicinity of dislocation cores for smaller *α* was observed (Supplementary Figs 14,15). Calculations indicate the bands near the Fermi level (long-dashed red line) are almost flat with negligible dispersion for all three angles, indicating strong Fermi level pinning. The first two bands above the Fermi level (red line) come mostly from the Mo 4d orbital at the 5–7 dislocation cores (‘Down' core for the first band and ‘Up' core for the second band). These energy levels near the Fermi level may serve as resonant charge-scattering centres, a notion that can be similarly surmised from the strongly localized charges at the GBs (Fig. 4c, ‘Down' core at the centre and ‘Up' core at the right edge of the supercell). In contrast, at the mirror boundary, the Fermi level crosses the completely delocalized state in the middle of (Γ–Y), revealing metallic character due to the 4-4 ring dislocation cores (Supplementary Fig. 16). The delocalized metallic feature with weak pinning provides a likely explanation for the high mobility of the mirror GBs.

Angle-dependent transport was then modelled by considering local band bending at the boundary induced during charge equilibration, as shown schematically in Fig. 4d. At equilibrium, the interfacial *Φ*_b_ is approximated by *Φ*_b_=*W*_GB_−*W*_MoS2_−*E*_d_, where *W*_GB_=*E*_c_+*E*_F_ is the GB work function, *W*_MoS2_ is the MoS_2_ work function and *E*_d_ is the trap energy with respect to the GB work function. *E*_d_ is fixed due to Fermi level pinning, resulting in the equilibrium band diagram displayed at the bottom of Fig. 4d. The work functions for the three GB structures obtained by DFT are 5.67 eV (7°), 5.71 eV (13°) and 5.50 eV (22°). Due to the presence of band-tail dispersion in the pristine MoS_2_, *W*_MoS2_ is unknown and *V*_gs_-dependent, and an exact *Φ*_b_ is undefined. However, relative values of *Φ*_b_ are compared with the JWKB experimental results in Fig. 3c; an offset of 150 meV was added to the calculated values. Both the ‘Up' and ‘Down' dislocation types show nearly identical trend, with *Φ*_b_ decreasing at higher *α*. The localization of charges at the boundary and subsequent Fermi level pinning determine the effective GB potential barrier height. GBs with defect states that are energetically farther from the conduction band (smaller *E*_d_) will have greater *Φ*_b_ values. Consequently, in MoS_2_ the exact GB atomic structure determines the barrier, and, although the density of dislocation cores increases with *α,* the charge density per unit length actually decreases as dislocation density increases. These results indicate that boundary transport is consistent with the previous theoretical treatment of 2D systems, although no exploration of the exact scattering mechanism at the GB is possible here.

For incoherent HA-GBs, more complicated defects than 5–7 cores exist, along with a greater number of dangling bonds in a zigzag shape or containing holes (Fig. 2), which cannot be explained by our JWKB tunnelling barrier treatment. As opposed to a straight potential barrier found in coherent GBs, a non-periodic kinked boundary likely forms a wider depletion region with regions of greater localized charge density. Furthermore, due to growth limitations for LA-GBs (<7°), we were unable to characterize the transport properties for very small *α*. In this regime, the induced potential perturbations from individual dislocation cores will become more isolated as *α* decreases. We postulate that carriers will simply pass within the sawtooth-like GB potential, leading to improved mobility with reduced *α*.

In conclusion, we have provided a more unified picture of the relationship between mobility, merging angle and atomistic structures of the GBs of monolayer MoS_2_. The results provide practical expectations regarding transport properties in large-area films, which will be restricted largely by the poor mobility across LA GBs. The results obtained in this work are applicable to other similar 2D systems, and contribute to the fundamental understanding of transport in semiconductors.

## Methods

### MoS_2_ grown on SiO_2_/Si wafer by CVD

OptiPrep density gradient medium (Sigma-Aldrich, D1556, 60% (w/v) solution of iodixanol in water) (defined as solution A) was purchased and used as a medium to dissolve promoter and precursor. It has no influence on growth. The promoter was prepared by dissolving sodium cholate (SC) hydrate (Sigma-Aldrich, C6445) in deionized (DI) water (0.6 g of SC in 30 ml of DI water), defined as solution B. A solution-phase ammonium heptamolybdate (AHM) precursor was also prepared by dissolving ammonium molybdate tetrahydrate (Sigma-Aldrich, 431346) into DI water (defined as solution C). The prepared 0.2 mM AHM precursor was mixed with SC promoter in DI water and then spin-cast on SiO_2_/Si wafer. Prior to the spin-casting process, the solutions were mixed in the ratio A:B:C=0.55:3:1. The mixing order (A→B→handshake→C) is critical to prevent micelle generation. The solution was dropped onto SiO_2_/Si wafer and followed by spin-casting at 2600, r.p.m. for 1 min. A two-zone CVD system was used to control sulfur (zone 1) and substrate (zone 2) efficiently. Here, 200 mg of sulfur (Sigma, 344621) was loaded into zone 1, while the substrate-containing metal precursor was placed at zone 2. All growth in this work was carried out at atmospheric pressure. For growth, zone 1 was heated up to 210 °C at a rate of 42 °C min^−1^. In the meantime, the temperature of zone 2 (growth temperature) were set to 780 °C.

### Transfer of MoS_2_ for device fabrication or TEM grid

Poly(methyl methacrylate) (PMMA A4, Chem) was spin-coated onto the as-grown MoS_2_ (2000, r.p.m., 1 min) as a holder and protective layer with no post-annealing step. The MoS_2_ and PMMA support were then detached from the SiO_2_/Si substrate by floating the PMMA/MoS_2_/SiO_2_/Si, with the PMMA side up, in a hot 2-M NaOH solution. Samples grown on SiO_2_/Si were floated for less than 1 min. Next, the PMMA/MoS_2_ was washed by transferring the structure into DI water (4 times). Finally, the PMMA/MoS_2_ was scooped out in pieces and placed on either a Quantifoil TEM grid with a gold-supported thin film (PELCO, 200 mesh, gold, 1.2-μm holes) or a SiO_2_/Si wafer for device fabrication. The grids were stored under ambient conditions overnight to increase adhesion between the PMMA/MoS_2_ and the grid. PMMA was ultimately removed by vapour acetone. The grids were then annealed at 180 °C in a vacuum chamber at high pressure (∼7.5 × 10^−5^ Torr) for at least 12 h prior to TEM analysis. For device fabrication, the MoS_2_ was dried at 80 °C for 5 min. Before removal of the PMMA, the entire sample was immersed in DI water for a few seconds so as to wash away residual NaOH solution that remained on the surface of the PMMA during the detaching step. The sample was subsequently immersed in acetone for a few minutes to remove the PMMA, followed by cleaning in isopropyl alcohol.

### Device fabrication

The synthesized MoS_2_ on an SiO_2_/Si substrate was transferred onto a highly doped (10^19^ cm^−3^) silicon substrate with a 100-nm-thick thermal oxide layer by a conventional transfer method mentioned in the previous TEM sample preparation. Glovebox preparation was employed for baking samples at 120 °C for SiO_2_ and MoS_2_ surface trap passivation for 2 h before beginning fabrication. All further baking steps during fabrication were also performed in the glovebox. Electron beam lithography and evaporation (Ti/Au 7/35 nm) were used to define the MoS_2_ electrode contacts for each sample. MoS_2_ was patterned into an elongated Hall bar structure via a final electron beam lithography step, followed by a weak 10-W plasma etch for 10 s under a 30-sccm flow of SF_6_.

### Transfer measured device to a TEM grid

Electrically characterized devices were transferred onto TEM grids to confirm the misorientation angle (SAED patterns) and the atomic structure of each GB (high-resolution STEM images). It is important to note that we succeeded in transferring the entirety of each device, which allowed for a direct investigation of the electrical behaviour and its correlation to both the atomic structure of the GBs and defects in MoS_2_. Here, the transfer process was similar to the one previously mentioned. However, the method employed in this study is compatible with Ti/Au electrodes. While Ti is not the optimum contact for MoS_2_, it is more easily transferred, as the Ti layer is easy to detach from the SiO_2_/Si substrate compared to other metals such as Cr. The transfer time for this process is longer than that associated with typical transfer procedures, which require less than a minute to detach the PMMA/MoS_2_ from the SiO_2_/Si substrate.

### TEM and DF-TEM

TEM imaging was conducted using a JEM ARM 200F machine operated at 80 kV; no damage was apparent in the samples at this accelerating voltage. The acquisition time for DF imaging was 1 s using the smallest objective lens aperture. The HR-TEM imaging acquisition time was also 1 s.

### ADF-STEM

ADF-STEM imaging was conducted using a probe aberration-corrected JEM ARM200F operated at 80 kV. High-angle ADF images were acquired at 20 mrad convergence angle. Because the energy of the low-voltage electron beam was below the damage threshold energy, the pristine MoS_2_ lattice remains stable and defect-free with regular scanning. Images presented in the main text were acquired with a medium-angle ADF detector with a collection angle ranging from 50 to 180 mrad and an acquisition time of 40 μs per pixel. We have used controlled imaging time (within 1 min) under high-magnification STEM node to avoid beam damage on the MoS_2_ sample^6^,^24^,^25^,^26^.

### Density functional calculations

DFT calculations performed with DMOL^3^ code^27^,^28^ were used to study the geometric structures and electronic states of different MoS_2_ monolayers. Geometries were fully optimized within a local spin density approximation with the Perdew−Wang correlational (LDA/PWC)^29^ until the convergence criteria for energy, force and displacement were less than 10^−5^ Hartree, 4 × 10^−3^ Hartree Å^−1^ and 5 × 10^−3^ Å, respectively. All-electron basis sets were utilized for all elements. A Monkhorst–Pack sampling scheme with a 2 × 2 × 1 *k*-point mesh was used for geometrical optimizations, and a 4 × 8 × 2 *k*-point mesh was employed for electronic structure calculations. All models for structure optimization were supercells with a sufficiently large size and minimal defect interactions. The super sizes of all GB types were the same, as shown in Fig. 4c. For example, the supercell size is 43 × 18 × 20 Å for the 7° GBs.

## Additional information

**How to cite this article:** Ly, T. H. *et al.* Misorientation-angle-dependent electrical transport across molybdenum disulfide grain boundaries. *Nat. Commun.* 7:10426 doi: 10.1038/ncomms10426 (2016).

## Supplementary Material

Supplementary Information Supplementary Figures 1-16, Supplementary Notes 1-3, Supplementary Methods and Supplementary References.
