# Supplementary material for: Misorientation-angle-dependent electrical transport across molybdenum disulfide grain boundaries
Source: Nat Commun. 2016 Jan 27;7:10426. doi: 10.1038/ncomms10426 (PMC4737806; doi:10.1038/ncomms10426)
Supplement: Supplementary Information — Supplementary Figures 1-16, Supplementary Notes 1-3, Supplementary Methods and Supplementary References. [file ncomms10426-s1.pdf]

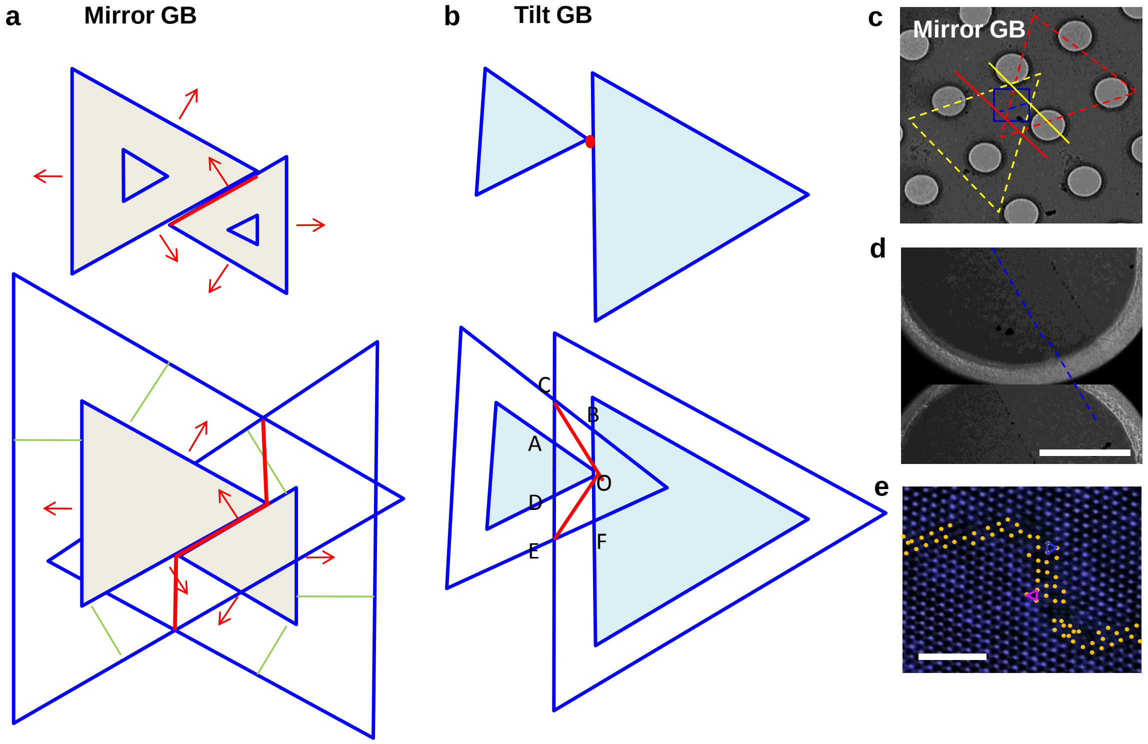

Supplementary Figure 1 | Schematic showing a simple way to define the GB position of merged MoS_2 flakes based on growth kinetics. a, Mirror GB and **b,** Tilt GB. Assume that the growth speed of each edge is approximately the same, because all the edges are zigzag types. For mirror GBs, the two grains are nucleated, meet each other and one section of GB (red line) is naturally formed at this time. The two grains continue to grow at the same speed, which means that the six green line sections have the same length. At last, the two more GB (red lines) will be formed from simple geometry. For tilt GBs, the meeting point during growth and the straight lines (two red lines) need to be defined. The GBs are both the middle-angle lines. If the growth speed of each edge is equal, then: <ACO=<BCO, <DEO=<FEO (< is the angle notation). Therefore, the way to define the low-angle tilt GB is even easier, just drat two lines directly which divide the two angles. However, the above method assumes that the edges of the grains grow at the same speed (near equilibrium conditions). The kinetic factors like the transportation of sources take effect during real growth made the straight line bended. Here, the analysis is based on kinetics, thermodynamic points aren’t included. **c,** TEM image of transferred MoS_2 on TEM grid, red and yellow triangles marked the grown flake in (a). **d,** DF-TEM image acquired in

the region indicated by the blue square in (c)**,** shown the straight GB (blue line) with a lot of holes; scale bar is 500 nm. **e,** HR-STEM image shown the atomic structure of mirror GB, with continuous 4-4 cores; scale bar is 200 nm.

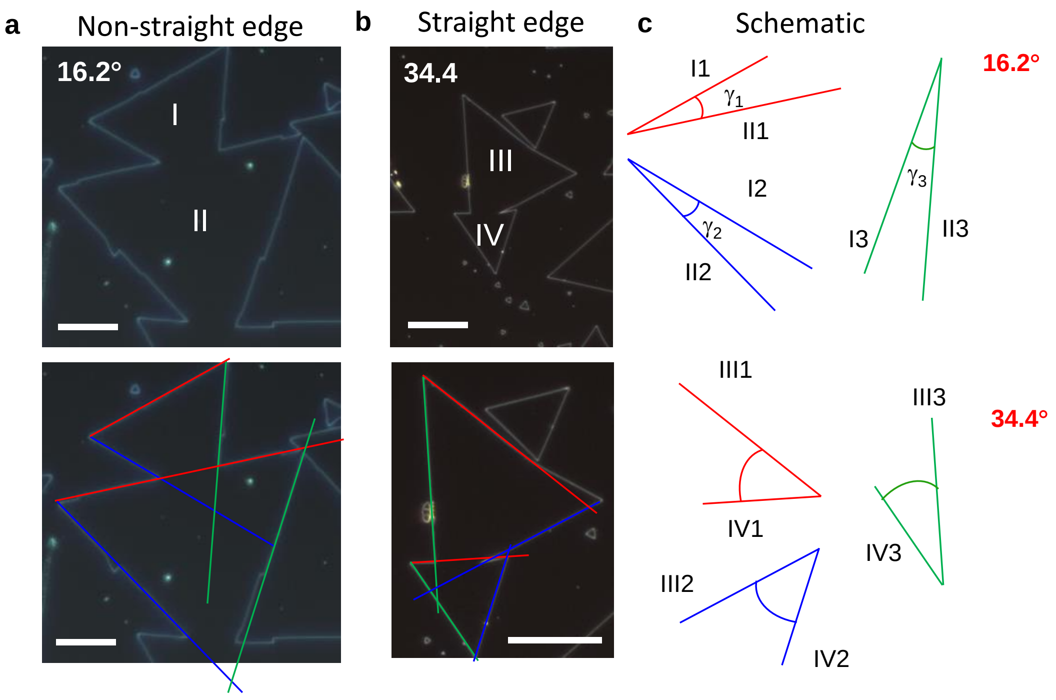

$$Misorrientation\ angle = \frac{<\gamma_1 + <\gamma_2 + <\gamma_3}{3}$$

Supplementary Figure 2 | Misorientation angle between two domains defined on an OM image by the edge alignment of triangles. OM image of **(a)** a non-straight edge sample and **(b)** a straight edge sample**.** Due to the atomic steps or kinks, the edges of these triangles deviated from the zigzag (Mo-terminated) edges. Firstly, we can skip the kinks on the edges of (a)**,** and simply select the section right before or after the kink. Secondly, when the triangles are not exactly equatorial triangles (some of the angles are not quite 60°), we use the three sets of edge pairs from the two triangles **(c)** to measure the misorientation angles and then average the results to minimize error. Scale bar is 10 μm.

a b

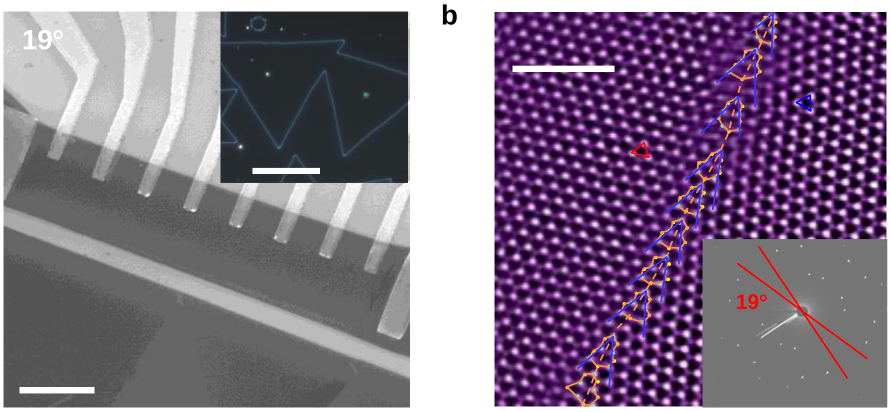

Supplementary Figure 3 | Standard error for misorientation angle between two domains by the edge alignment of triangles using optical microscopy, compared with SAED pattern and HR-STEM observation. a, SEM image of devices on SiO_2/Si substrate; scale bar is 3 µm. The inset shown a dark field optical image of two triangles merged with a 19° tilt angle; scale bar is 20 µm. While the ideal case for MoS_2 flake growth is the forming of an equilateral triangle, most of the flakes used in our experiments are not equilateral triangles. The misorientation angle from these flakes was defined following Supplementary Fig. 2; the angle is 19°. After electrical measurement, the device was transferred to a TEM grid to confirm the misorientation angle between the two flakes. **b,** High-resolution STEM image of the GB between two flakes; each domain is marked by red and blue triangles, which correspond to three Mo atoms location. The angle difference between the red and blue triangles indicates the misorientation angle between these two flakes is ~ 19°, which was consistent with the SAED pattern (inset image).

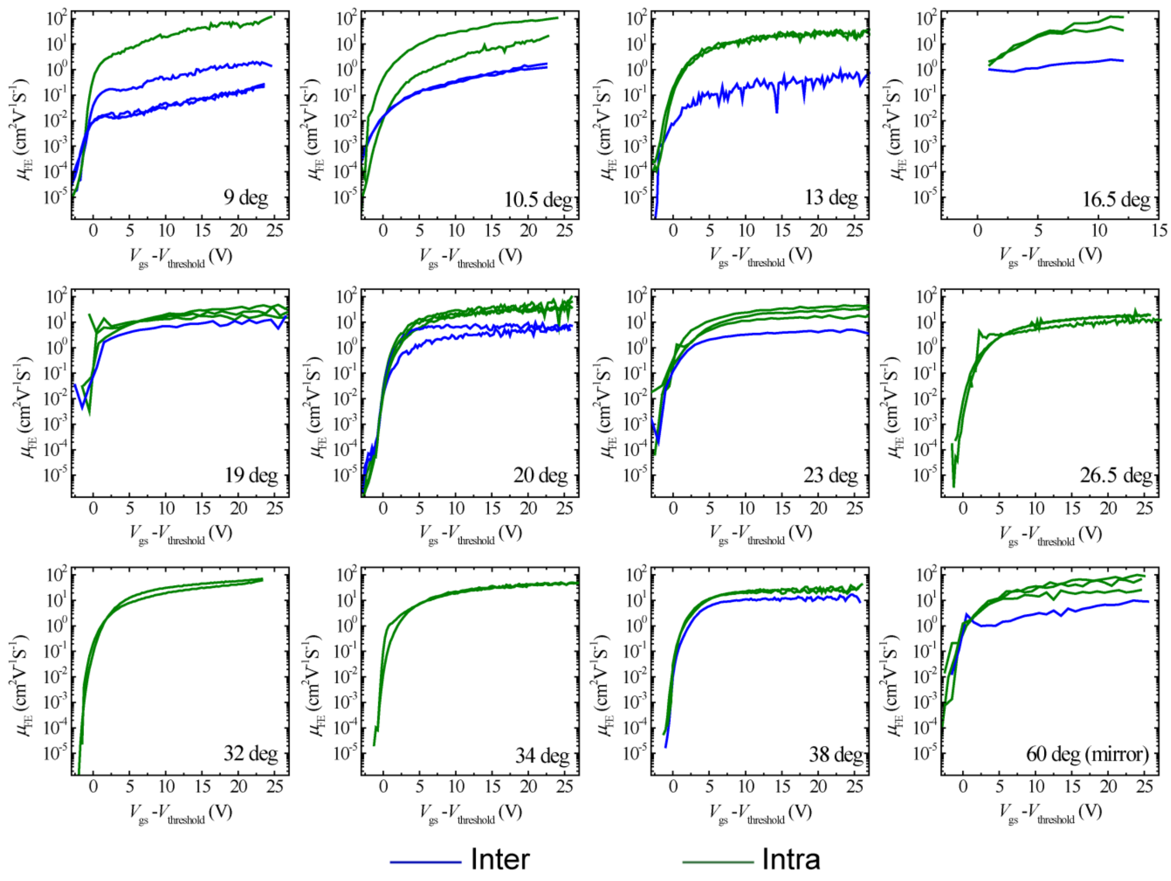

Supplementary Figure 4 | 4-terminal mobility data calculated from 300K I_{sd}-V_{gs} data via $\mu_{FE} = Wg_m / LV_{sd}C_{SiO_2}$ with W defined by the total width of the MoS_2, L defined by the length of the gap between respective electrodes plus the width of one electrode (e.g. due to ½ width from each electrode), V_{sd} is the measured voltage difference on the respective probes, and C_{SiO2} = 35.1 nF cm^{-2} for 100 nm thick SiO_2. The L and W values were chosen for consistency among all samples, although the definition likely slightly underestimates μ_{FE}.

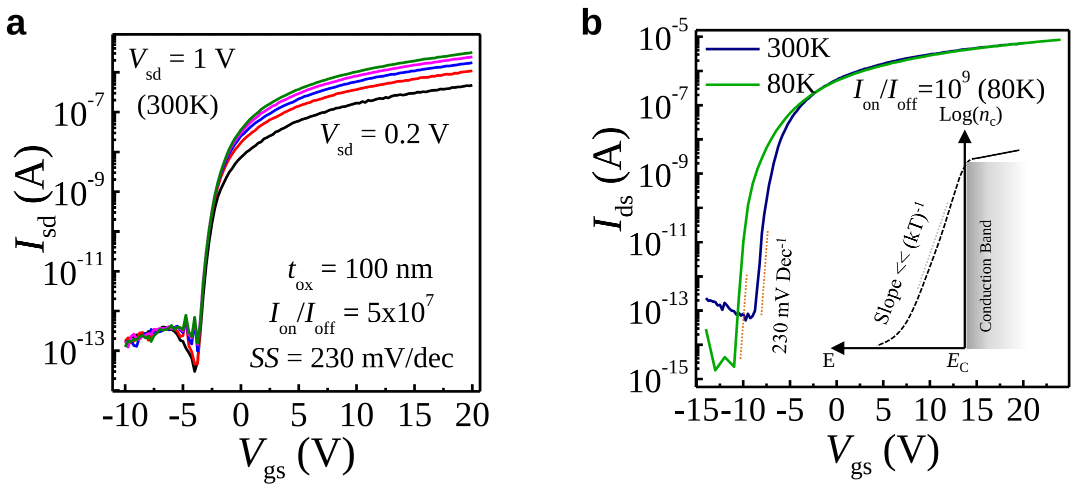

Supplementary Figure 5 | a, 2-terminal intra-domain I_{sd}-V_{gs} data at various V_{sd} potentials (0.2, 0.4, 0.6, 0.8 and 1V). All samples in this work were limited by a 230 mV/dec subthreshold swing for the 100 nm SiO_2 gate dielectrics. **b,** The subthreshold swing was also temperature independent, indicating that band-tail state dispersion limits transport properties.

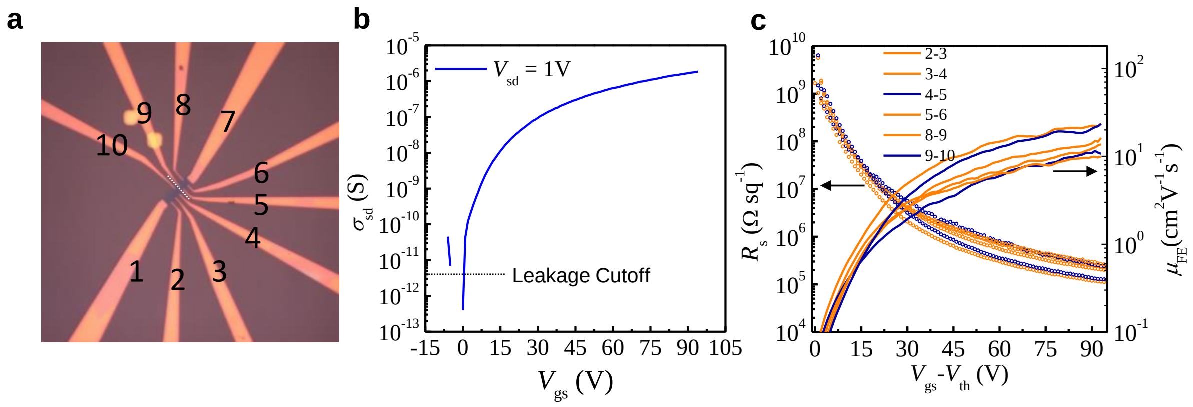

Supplementary Figure 6 | Highest performing devices fabricated outside a glovebox. a, Optical image of MoS_2 devices on SiO_2 (300 nm)/ Si substrate. The GB location, marked with a white dot line, crossed 2 pairs of electrodes (4-5 and 9-10). **b,** Two terminal I_{ds}-V_{gs} data from a pair of electrodes in (a)**. c,** Mobility (solid lines) and R_s from the device in panel (a). Similar to previous work, performance in the intra/inter-domain regions is indistinguishable and limited to μ_{FE} < 25 $cm^2V^{-1}s^{-1}$ and R_s > 100 kΩ/sq for all samples. It is important to note that this range is identical to that obtained for inter-domain only results for glove box-fabricated samples, indicating that ambient preparation methods are unsuitable for 2D fabrication and will likely generate nontrivial densities of vacancies and other short-range scattering centres.

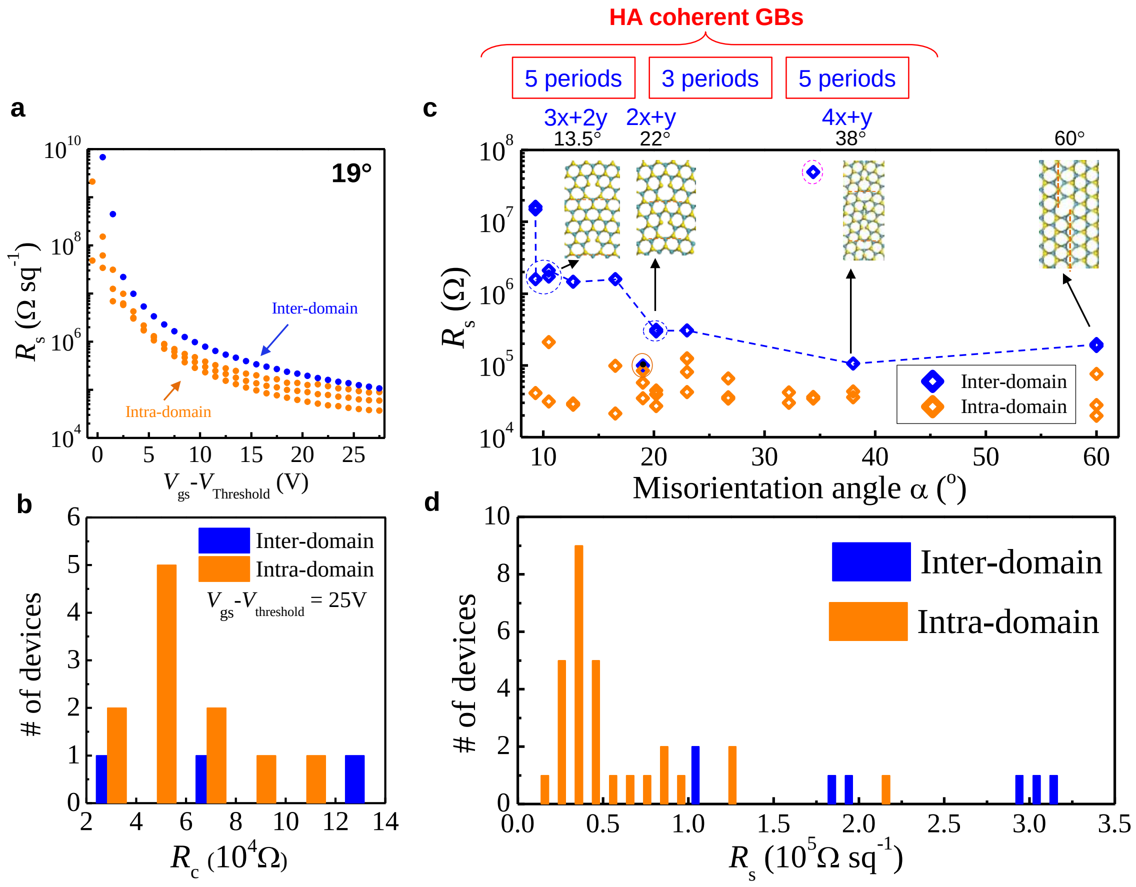

Supplementary Figure 7 | R_s and R_c with respect to the GB misorientation angles. a, R_s for the 19° device was measured as a function of V_{gs}. **b,** Contact resistance of the devices with a mean R_c = 52.45 kΩ ± 16.5 kΩ and no intra/inter-domain dependence. **c,** R_s exponentially increases as the misorientation angle increases. An unexpectedly small R_s is measured for the 19° region due to the position of the grain boundary, as described in the main text. The 34° boundary was poorly connected, and only survived a single measurement, and does not reflect intrinsic boundary properties. For coherent GBs, a two order of magnitude decrease in R_s was observed for misorientation angles between 9° and 60°. Atomistic models are reflective periodic dislocation cores for different misorientations as observed from STEM/TEM. **d,** Histogram of the data in panel (c). Only the 7 lowest R_s values for the inter-domain are shown, as a large order-of-magnitude difference skewed the visualization when all data were displayed. The median of the intra-domain R_s was 39.4 kΩ.

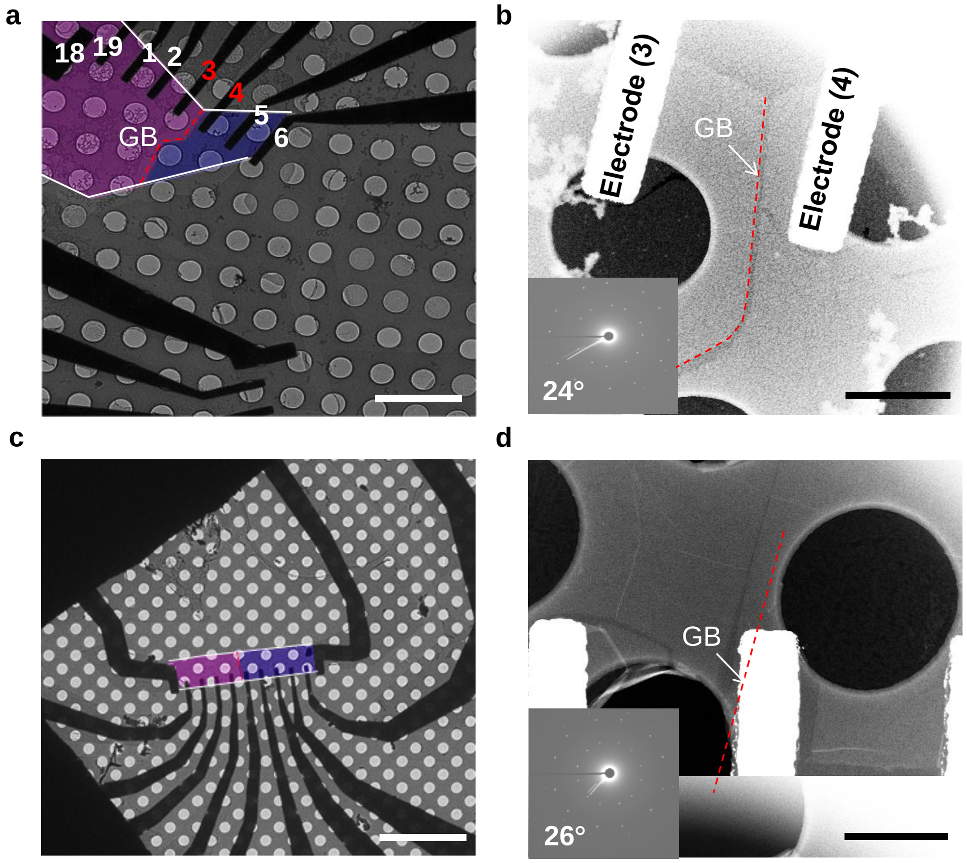

Supplementary Figure 8 | TEM observations of certain devices showed that the flakes were indeed separated. a, TEM image of the device was transferred onto a TEM grid, each electrode is marked with a different number, and the GB is located between electrodes 3 and 4; scale bar is 5 µm. **b,** DF-TEM image showing that the GB was broken after transferred and thus, no HR-STEM data was obtained; the inset reveals that the angle different is roughly 23°; scale bar is 1 µm. **c, d,** The 26° device showing characteristics similar to those in (a, b). Scale bar are 10 µm and 1 µm in (c,d).

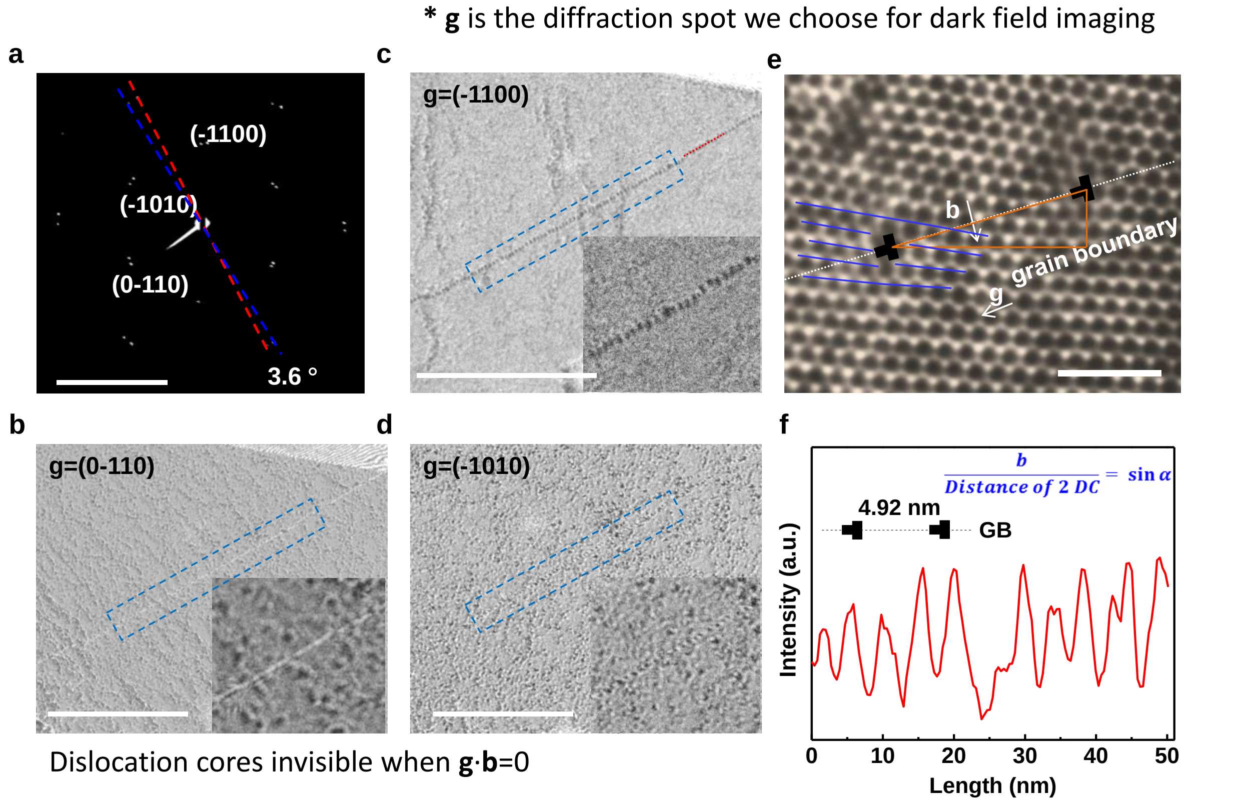

Supplementary Figure 9 | Correlation of dark field TEM images, Burger vector, periodicity, and LAGB theory. a, SAED pattern showing that the angle between two grain domains is approximately 3.6°, as denoted in the angle between two dashed blue lines; scale bar is 5 1/nm. **b-d,** Dark field images of the grain boundary correlated with each GBs vector ***g*** = (0-110), (-1100), and (-1010) in (a). The inset displays an enlarge image of the dash blue square. Scale bar are 200 nm in (b,c) and 100 nm in (d). No dislocation core was observed when the selected diffracted beam is on vector ***g*** = (0-110). **b,** because the Burgers vector is (-2110), fulfill the dislocation cores invisible law when ***g.b*** = 0 (***b*** is the Burgers vector of the dislocation). When ***g.b*** ≠ 0, **(c, d)**, dislocation cores appeared as the black dots on dark field image. **e,** Atomic structure HR-STEM image of 3.6° low-angle tilt GBs; the dislocation cores are denoted by the Burgers vector and the solid blue line. **f,** Distance between each dislocation core of the red line in **c;** this distance is similar to that between the two dislocation core in (e). The distance between two dislocation cores is also consistent with low angle GB theory, as shown by the blue function in the inset of (f).

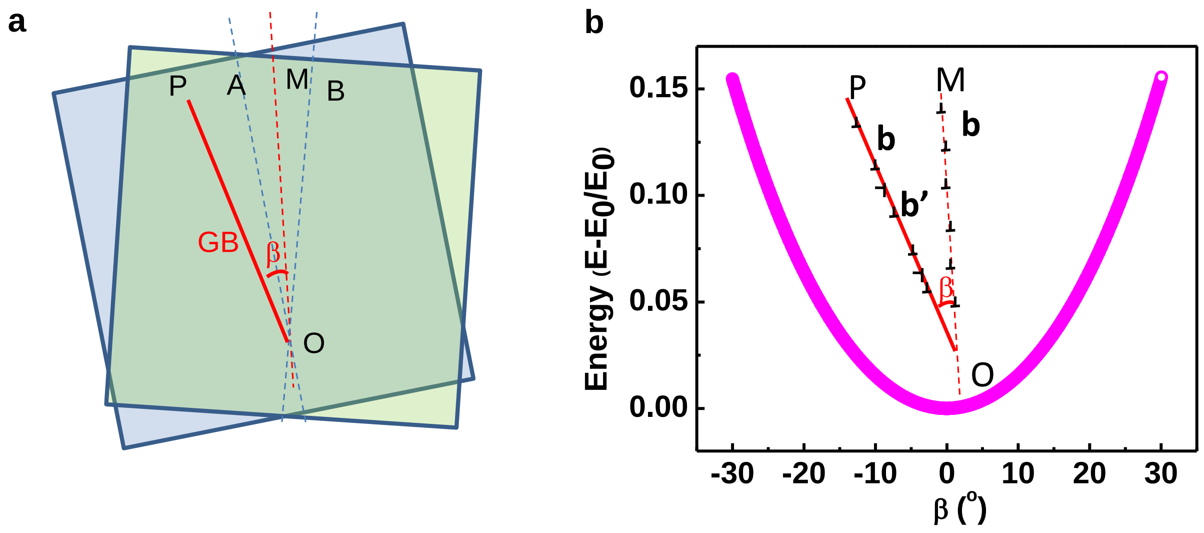

Supplementary Figure 10 | Energy of LA-GB along different directions with respect to the crystal. **a,** The two squares denote two different grains; OA and OB represent the same crystal direction (armchair direction) of two MoS_2 grains, while OM is the semi-angle of <AOB. GB (red solid line) with an angle from the OM is defined as β (different from the misorientation angle α). **b,** Energy of the LA-GB, with the inset showing the difference between the angle of the GB and the semi-angle of <AOB. Assuming that the Burgers vector on the original OM is ***b***, the Burgers vector on OP is then ***b*** combined with ***b'***. The effective **B**urgers vector on line OP is $\boldsymbol{b}/\cos\beta$. Thus, the GB energy per length is

$$E \sim (\boldsymbol{b}/\cos\beta)^2 * \cos\beta$$

where $\boldsymbol{b}/\cos\beta$ is the energy of a single dislocation and cosβ is the density of dislocations per unit length. So $E \sim (\cos\beta)^{-1}$. Therefore, the lowest energy angle is located near the armchair directions, and the straight line is energetically favored.

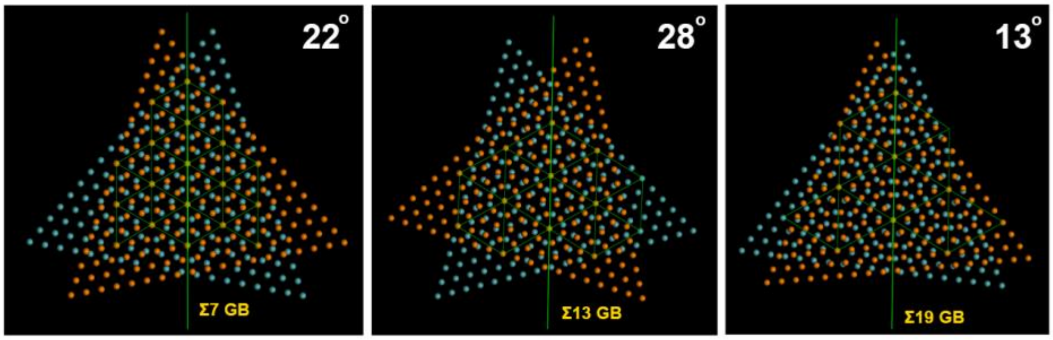

Supplementary Figure 11 | The reciprocal of the ratio of coincident sites between two adjacent grains or domains is defined in coincident site lattice (CSL) theory. For this reason, we present Σ7, Σ13, and Σ19 as the three main coherent boundaries.

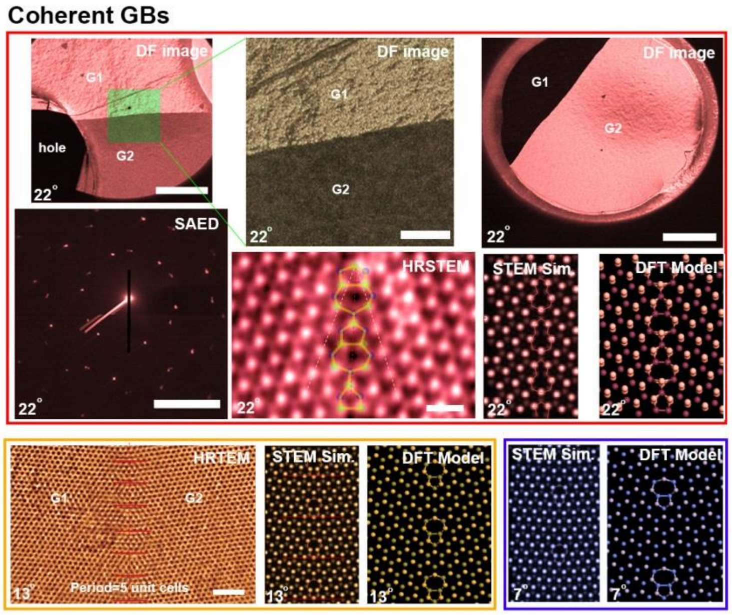

Supplementary Figure 12 | TEM, DFT calculations and STEM image simulations for high-angle coherent boundaries (13°, 22°) and low-angle boundary (7°). The periodicities for 7°, 13° and 22° boundaries are 7, 5, and 3 unit cells, respectively. Scale bar are 200 nm in the left and right DF images. The middle DF image, extended for the green marker of the left image has scale bar of 50 nm. Scale bar are 0.5 nm for HRSTEM image of 22° GB and 2 nm for HRTEM image of 13° GB.

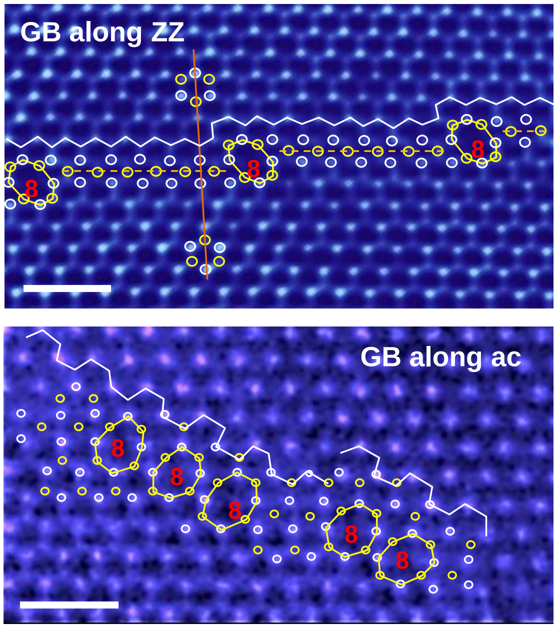

Supplementary Figure 13 | Structure of mirror GBs. a, GB that follows a zigzag direction and is composed of 4-4 cores forming a straight line with kinks by the 8 cores. **b,** GB that follows the armchair direction and is composed of 8 dense cores. Scale bar is 1 nm.

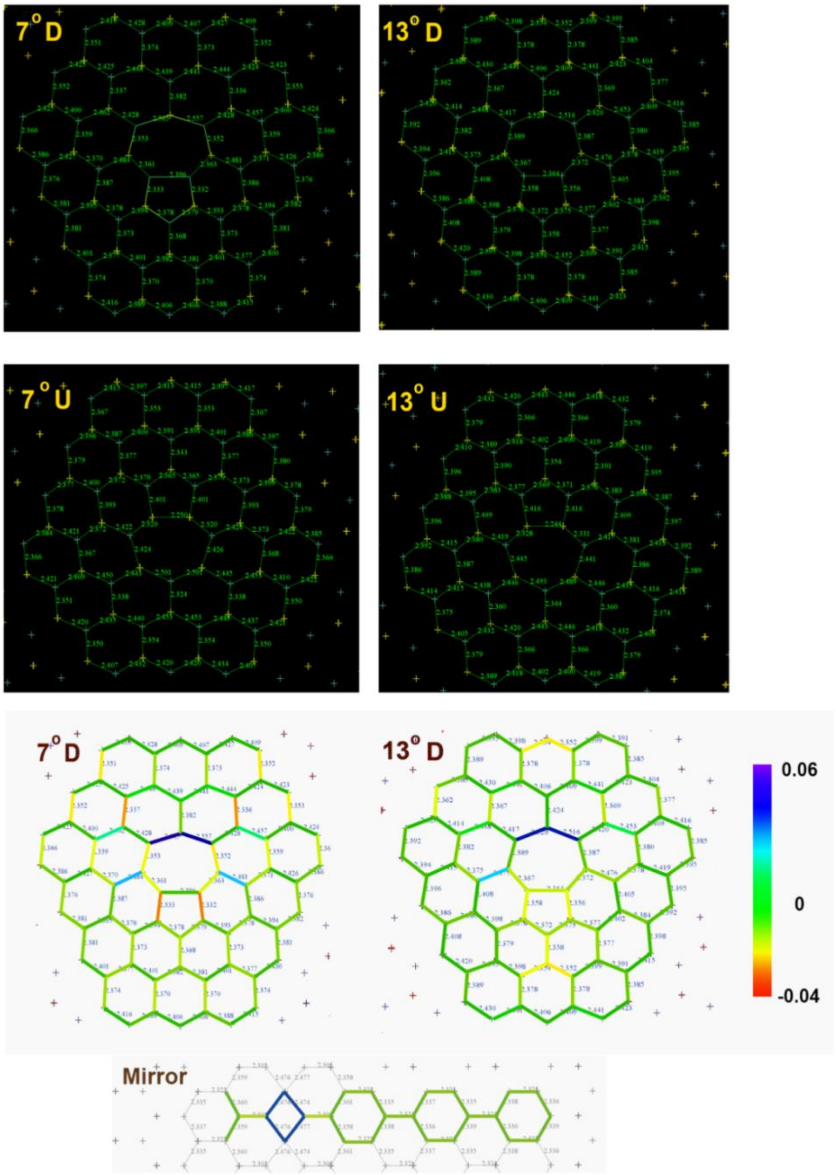

Supplementary Figure 14 | The DFT calculated atomic models for 7° (‘Up’ dislocation cores) and 13° (‘Down’ dislocation cores) misorientation angle GBs; the supercell sizes are greater than 6nm×6nm×2nm which is sufficiently large. Each Mo-S bond length is highlighted. If we set the equilibrium bond length to 2.4 Å, elongation or shrinking of the bond length can be mapped below. This mapping shows that the effect of strain around the higher angle (13°) GB dislocation cores is smaller than that around the lower angle (7°) GB dislocation cores for both ‘Up’ and ‘Down’-type dislocations. This trend is consistent with theoretical results, which show that the energy per dislocation core in lower angle GB is larger[1]. The bond length strain effect around a 4-4 mirror GB is also presented, and a smaller strain effect than that around the 5-7 dislocation cores is observed.

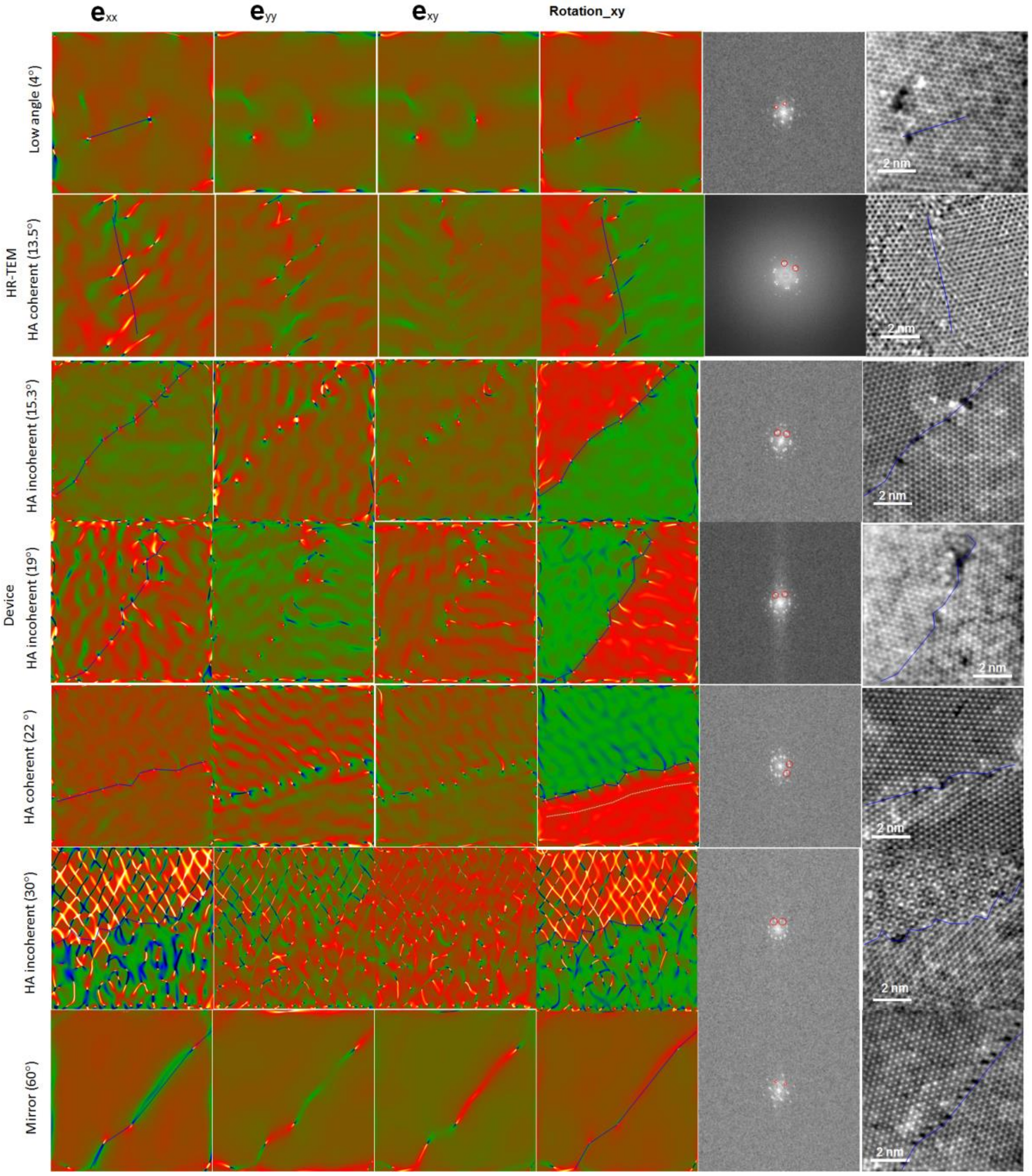

Supplementary Figure 15 | Strain-field mapping of different misorientation-angle GBs using GPA strain mapping from HR-STEM images. The strain color scale corresponds to a range from -1 (green) to +1 (red), with the boundary between red and green denoted by a blue line. From 4° (low angle) to less than 60°, the higher misorientation angle GBs show strain field with larger e_{xx}, e_{yy}, e_{xy} and rotation lattice ($rotation_{xy}$). For the 60° (mirror) GB, the strain field is located right at the GB.

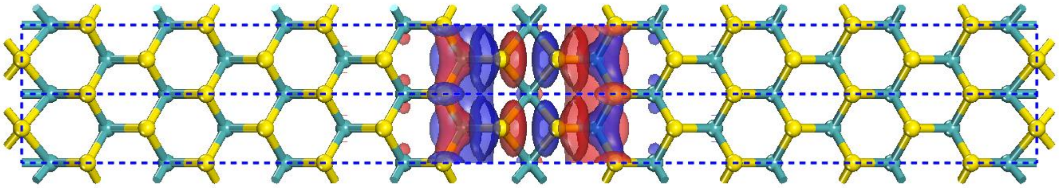

Supplementary Figure 16 | The partial charge distribution induced by the defective atoms at the midgap states on mirror GB.

Supplementary Note 1 | Band-tail state description

Using Fermi-Dirac statistics and setting the conduction band edge as a reference ($E_C = 0$), the delocalized charge density associated with transport at the conduction band edge in n-type MoS_2 is described by the following exponential relationship:

$$n_{\mathrm{band}} = N_{\mathrm{band}} \exp\left(E_{\mathrm{F}}/kT\right) \tag{1}$$

where N_{band} is the band-edge charge density. However, the band edge mobility in MoS_2 is far less than that expected for a perfect lattice with phonon-limited scattering[2] (μ_{band} ≈400 $\mathrm{cm^2V^{-1}s^{-1}}$). Due to the temperature-independent nature of the *SS* (~230 mV/dec) (Supplementary Fig. 4), a large concentration of localized charges (n_{loc}) associated with a band-tail states exists in MoS_2 and thus, $n_{\mathrm{loc}}/n_{\mathrm{band}} >> 1$ for applicable experimental conditions. The percentage of this localized charge that participates in transport is negligibly small and limits carrier mobility. In this case, the effective field-effect mobility is[3]:

$$\mu_{\mathrm{FE}} = \mu_{\mathrm{band}}\left(1 + n_{\mathrm{loc}}/n_{\mathrm{band}}\right)^{-1} \tag{2}$$

By taking the natural log and substituting $n_{\mathrm{loc}}/n_{\mathrm{band}} >> 1$, Eq (2) simplifies to:

$$\ln\left(\mu_{\mathrm{FE}}\right) - \ln\left(\mu_{\mathrm{band}}\right) = -\ln\left(n_{\mathrm{loc}}/n_{\mathrm{band}}\right) \tag{3}$$

Localized band edge states are also modeled by an exponentially decaying density of states:

$$n_{\mathrm{loc}} = N_{\mathrm{defect}} \exp\left[\left(E_{\mathrm{F}} - E_{\mathrm{defect}}\right)/\beta_1\right] \tag{4}$$

where N_{defect} is the local defect density, E_{defect} is the defect level with respect to the conduction band edge, and $\beta_1 >> kT$ describes the exponential decay of band tail states for a specific device.

For a single device, the above relationship holds true. However, the defect density is likely not constant both within a flake and when comparing different flakes in spite of special care taken to reduce sample variation. The defect density depends on the growth, transfer, and fabrication procedures. Similarly, substrate disorder should also have a small spatial dependence

on the same substrate and for devices on different substrates. The impact of the spatial inhomogeneity is accounted for by assuming a normal distribution of the quantities E_{defect} and β_1. To introduce this statistical distribution of spatially inhomogeneous defects (which are further related to statistical mobility distribution), $\left(E_{\text{F}} - E_{\text{defect}}\right)/\beta_1$ can be replaced with an arbitrary normal distribution function. As a consequence, Eq. (4) is replaced by:

$$\bar{n}_{\text{loc}} = N_{\text{defect}} \exp\left[g\left(E_{\text{F}} - E_{\text{defect}}\right)/\beta_1\right] \qquad (5)$$

Eq. (5) represents a statistical distribution of the localized defect density, where $g\left(E_{\text{F}} - E_{\text{defect}}\right)/\beta_1$ is a normal distribution function of defects. Upon substituting Eq. (5) into Eq. (3), we obtain the log-normal distribution of the mobility:

$$\ln\left(\bar{\mu}_{\text{FE}}\right) - \ln\left(\mu_{\text{band}}\right) = -\ln\left(N_{\text{defect}}/n_{\text{band}}\right) + g[\left(E_{\text{F}} - E_{\text{defect}}\right)/\beta_1] \qquad (6)$$

From Eq. (6) it can be seen that $\ln\left(\bar{\mu}_{\text{FE}}\right) \propto g\left(E_{\text{F}} - E_{\text{defect}}\right)$, as all other parameters are constant or vary trivially due to the natural logarithm. While $g\left(E_{\text{F}} - E_{\text{defect}}\right)$ was defined as normal distribution, this function should at least be symmetric about an expected/mean value. This must be true in order for the experimental data to be statistically meaningful due to acceptable sample reliability, consistent fabrication, and processing conditions. A Gaussian function is a good example for simplicity, but the specific type of symmetric distribution is not critical here.

If band-tail states are the limiting factor for transport, a large set of devices should then have a measured μ_{FE} that follows the $\bar{\mu}_{\text{FE}}$ dispersion in Eq. (6), e.g. a log-normal distribution. This is precisely what was observed in Fig. 1D in the main text. With an expected mobility of $\mu_{\text{FE}} = 44$ $\text{cm}^2\text{V}^{-1}\text{s}^{-1}$, $n_{\text{loc}}/n_{\text{band}} \sim 9$, which is consistent with one of the major initial assumptions when deriving the log-normal relationship. It should be noted that the inter-domain results do not fit within this log-normal distribution.

Supplementary Note 2 | Relating V_{th} to inter-domain charging

The presence of charge within the MoS_2 is due to intrinsic self-capacitance of the MoS_2 denoted by $C_{\mathrm{MoS_2}}$ and defect-related capacitance, C_{loc}. The local charge (n_{loc}) in the MoS_2 is therefore:

$$n_{\mathrm{loc}} = E_{\mathrm{F}}\left(C_{\mathrm{loc}} + C_{\mathrm{MoS_2}}\right) \tag{7}$$

At the peak *SS*, the relative change in the total charge is maximized when the Fermi level is varied. Consequently, the gate-induced charge (n_{gate}) and n_{loc} are minimized (zero). Since $|V_{\mathrm{gs}}| >> |E_{\mathrm{F}}|$:

$$n_{\mathrm{total}} = n_{\mathrm{loc}} + n_{\mathrm{gate}} = E_{\mathrm{F}}\left(C_{\mathrm{loc}} + C_{\mathrm{MoS_2}}\right) + \left(-V_{\mathrm{gs}}\right)C_{\mathrm{SiO_2}} = 0 \tag{8}$$

Here, V_{gs} at maximum *SS* is designated at V_{th} for purpose of analysis here, leading to the following expression:

$$E_F \frac{\left(C_{\mathrm{loc}} + C_{\mathrm{MoS_2}}\right)}{C_{\mathrm{SiO_2}}} = V_{\mathrm{gs}} = V_{\mathrm{th}} \tag{9}$$

As evident in Fig. 3A, no discernable differences in the peak *SS* result from the inter/intra-domain character. For the case of E_F within the band gap, the ratio of capacitances is a constant *k*:

$$\frac{\left(C_{\mathrm{loc}} + C_{\mathrm{MoS_2}}\right)}{C_{\mathrm{SiO_2}}} \approx \frac{C_{\mathrm{loc}}}{C_{\mathrm{SiO_2}}} = k \tag{10}$$

However, V_{gs} of the peak *SS* is not constant. Eq. (9) indicates that the values of E_{F} for intra-domain and inter-domain transport are not equal. Therefore $E_{\mathrm{F(inter)}} - E_{\mathrm{F(intra)}}$ is estimated by subtracting the median V_{th} for inter/intra-domain transport and plugging into Eq. (9):

$$V_{\mathrm{th(median,inter)}} - V_{\mathrm{th(median,intra)}} = \Delta V_{\mathrm{th}} = k\left(E_{\mathrm{F(inter)}} - E_{\mathrm{F(intra)}}\right). \tag{11}$$

Thus, a difference in the median V_{th} is a direct result of Fermi level variations in the two systems. The GB region is the primary source of mobility degradation in inter-domain transport and thus, the Fermi level difference results from the boundary region specifically. As a result, the GB is the source of an electrostatic potential boundary.

Supplementary Note 3 | MoS_2-Ti/Au Contacts

The Ti contacts utilized in the field-effect measurements were analyzed by comparing the results of 4-terminal with 2-terminal measurements. Contact resistance was roughly estimated by $R_C = R_{2T} - 2\alpha R_S$ with $\alpha = L/W$ as a geometric normalization parameter. The obtained results are shown in Fig. S6. The data are symmetrically distributed with an average of 52kΩ and no dependence on boundary position, as is expected. These resistances are orders of magnitude too large for practical applications, they do not influence the 4-terminal conclusions in this work. We note that recent progress in the field of local contact phase changes and the use of graphene or Al as contacts is promising in reducing these values[4, 5].

Supplementary Methods

Dark field optical microscopy (DF-OM). Optical microscopy (ZEISS, Axio Imager 2) was used to obtain images of the GBs surface morphologies of the transferred TMD samples.

Scanning electron microscopy (SEM). The surface morphology of the TMDs on the SiO_2/Si substrate was examined by field-emission scanning electron microscopy (FESEM) (JSM7000F, JEOL, Japan). An accelerating voltage of 15 kV was utilized to obtain a high contrast at different magnifications.

ADF image simulations. MoS_2 boundaries models and schematics were constructed with Materials Studio software. ADF image simulations were carried out using QSTEM with a probe size of 0.05 Å (spherical aberration coefficient, Cs =1 µm, Scherzer defocus = -2.5 nm).

Supplementary References.

1. A. P. Sutton, R. W. Balluffi. Overview no. 61 On geometric criteria for low interfacial energy. *Acta Mater.* **35**, 2177-2201 (1987).
2. N. Ma, D. Jena. Charge Scattering and Mobility in Atomically Thin Semiconductors. *Phys. Rev. X* **4**, 011043 (2014).
3. A. M. van der Zande *et al.* Grains and grain boundaries in highly crystalline monolayer molybdenum disulphide. *Nat. Mater.* **12**, 554-561 (2013).
4. X. Cui *et al.* Multi-terminal transport measurements of MoS_2 using a van der Waals heterostructure device platform. *Nat. Nano.* **10**, 534-540 (2015).
5. R. Kappera *et al.* Phase-engineered low-resistance contacts for ultrathin MoS_2 transistors. *Nat. Mater.* **13**, 1128-1134 (2014).
